# Add-On Effects of Chinese Herbal Medicine for Post-Stroke Spasticity: A Systematic Review and Meta-Analysis

**DOI:** 10.3389/fphar.2019.00734

**Published:** 2019-06-27

**Authors:** Yiyi Cai, Claire Shuiqing Zhang, Shaonan Liu, Zehuai Wen, Anthony Lin Zhang, Xinfeng Guo, Charlie Changli Xue, Chuanjian Lu

**Affiliations:** ^1^The Second Affiliated Hospital of Guangzhou University of Chinese Medicine (Guangdong Provincial Hospital of Chinese Medicine), the Second Clinical College of Guangzhou University of Chinese Medicine, Guangdong Provincial Academy of Chinese Medical Sciences, Guangzhou, China; ^2^China-Australia International Research Centre for Chinese Medicine, School of Health and Biomedical Sciences, RMIT University, Melbourne, VIC, Australia

**Keywords:** herbal medicine, meta-analysis, muscle spasticity, randomized controlled trial, stroke

## Abstract

**Background:** Treatment for post-stroke spasticity (PSS) remains a major challenge in clinical practice. Chinese herbal medicine (CHM) is often administered to assist in routine care (RC) in the treatment of PSS, with increasing numbers of clinical research and preclinical studies suggesting that it has potential benefits. Therefore, we conducted a systematic review and meta-analysis to evaluate the add-on effects and safety of CHM for PSS.

**Methods:** Five English and four Chinese databases were searched from their respective inception to 28 February 2018. We included randomized controlled trials that evaluated the add-on effects of CHM for PSS, based on changes in the scores of the (Modified) Ashworth Scale (AS or MAS), Fugl-Meyer Assessment of Sensorimotor Recovery (FMA), and Barthel Index (BI).

**Results:** Thirty-five trials involving 2,457 patients were included. For upper-limb AS or MAS, the estimated add-on effects of CHM to RC were significantly better when using oral (SMD −1.79, 95% CI: −3.00 to −0.57) or topical CHM (SMD −1.06, 95% CI: −1.40 to −0.72). For lower-limb AS or MAS, significant add-on benefits to RC were also detected (SMD −1.01, 95% CI: −1.43 to −0.59 and SMD −1.16, 95% CI: −1.83 to −0.49) using oral and topical CHM, respectively. For FMA and BI, better results were detected when adding CHM to RC, except for the subgroup of oral CHM for upper-limb FMA. Ten of the 35 included studies reported safety information, with two of them mentioning two mild adverse events.

**Conclusions:** Noting the quality concerns of the included trials, this review suggests that CHM appears to be a well-tolerated therapy for patients with PSS, and the potential add-on effects of CHM in reducing spasticity and improving the daily activities of patients with PSS require further rigorous assessment.

## Introduction

Spasticity can adversely impact almost half of stroke survivors (Watkins et al., 2002; Kwah et al., 2012; Zorowitz et al., 2013) and may worsen other post-stroke complications, including urinary and fecal incontinence, as well as skin infection (Bravo-Esteban et al., 2013; Martin et al., 2014; Gillard et al., 2015; Milinis and Young, 2015). In particular, spasticity can be a great barrier in rehabilitation for stroke recovery (Nair and Marsden, 2014).

Although there is uncertainty about the effects of specific rehabilitation interventions targeting post-stroke spasticity (PSS) and about the timing of their initiation, control of spasticity as soon as the patient’s posture or mobility is affected is generally encouraged (European Stroke Organization (ESO) Executive Committee and ESO Writing Committee, 2008; Miller et al., 2010; Smith et al., 2010; Stroke Foundation of New Zealand and New Zealand Guidelines Group, 2010; Chinese Society of Neurology and Stroke Prevention Project Committee of National Health and Family Planning Commission in China, 2012; National Institute for Health and Care Excellence, 2013; Nair and Marsden, 2014; Australian National Stroke Foundation, 2017). In terms of spasticity management, non-pharmaceutical intervention is preferred as first-line treatment; these include position management and manual stretching (Nair and Marsden, 2014). For some alternative therapies, such as shock wave stimulation, electrical stimulation, and repetitive transcranial magnetic stimulation, comprehensive assessment is required to confirm their effectiveness (Mally and Dinya, 2008; Stein et al., 2015; Dymarek et al., 2016a; Dymarek et al., 2016b; Dymarek et al., 2016c). When these therapies do not achieve a satisfying response, oral and invasive anti-spasticity medications could be considered (Bensmail et al., 2006; Bensmail et al., 2009; Harned et al., 2011). However, more than half of patients with PSS still suffer from moderate to severe disability after using current therapies (Sze et al., 2000), since the effectiveness is limited by a relatively short maintaining period, high costs, and unwanted adverse events, such as drowsiness and muscle weakness (European Stroke Organization (ESO) Executive Committee and ESO Writing Committee, 2008; Miller et al., 2010; Smith et al., 2010; Stroke Foundation of New Zealand and New Zealand Guidelines Group, 2010; Chinese Society of Neurology and Stroke Prevention Project Committee of National Health and Family Planning Commission in China, 2012; National Institute for Health and Care Excellence, 2013; Nair and Marsden, 2014; Australian National Stroke Foundation, 2017).

From a classical Chinese medicine perspective, PSS is also considered one of the clinical manifestations of stroke. The primarily etiology of PSS is a deficiency of *qi*, Blood, *yin*, or *yang*, that generates internal pathological products, such as Wind, Fire, Phlegm, or Stasis, blocking the meridian and collateral channels and resulting in the failure of nourishing tendons and muscles. Eventually, spasticity, limb stiffness, and contracture occur (Zhang and Xue, 2012). Therefore, Chinese herbal medicine (CHM) that could either restore the balance of *qi*, Blood, *yin*, and *yang* or clean up internal pathological products would be considered in the treatment of PSS (Zhang and Xue, 2012).

Nowadays, CHM is often administered in clinical practice as an adjunct to routine care (RC) for the treatment of PSS. Clinical research has also been increasingly conducted, with a focus on both orally or topically used CHM formulas, for PSS (Liu et al., 2014b; Zhu et al., 2014). Increasing numbers of preclinical studies have suggested that CHM single herbs and formulas are related to inhibition of certain types of neurotoxicity and certain anti-spasmodic activities (Hu et al., 2013; Huang et al., 2013; Li et al., 2014; Zhu et al., 2015).

In order to provide an overall evaluation of existing clinical evidence regarding CHM for PSS, we conducted a systematic review to address whether 1) CHM (including oral and topical CHM) in combination with RC (including pharmacotherapy and/or rehabilitation therapies) is more effective than RC alone in terms of spasticity severity, motor function, and activities of daily living; and whether 2) the use of CHM is safe.

## Methods

### Data Sources and Search Strategies

Five English databases (PubMed, Cumulative Index to Nursing and Allied Health Literature, EMBASE, Cochrane Central Register of Controlled Trials, and Allied and Complementary Medicine Database), four Chinese databases (the Wanfang Database, Chongqing VIP Database, Chinese National Knowledge Infrastructure, and Chinese Biomedical Database), and two online clinical trial registration websites (the International Clinical Trials Registry Platform and the Chinese Clinical Trial Registry) were searched from their respective inception to February 2017, with an updated search conducted in February 2018. Related trials and systematic reviews obtained by searching the references of the included studies were also researched. The detailed search strategy is presented in **Table S1**; three categories of search terms were used (“Chinese herbal medicine,” “post-stroke spasticity,” and “clinical trials”). Reporting details are available in File S1.

### Study Screening and Selection Criteria

Two researchers (YC and CZ) independently screened the titles, abstracts, and full texts to remove duplicates and irrelevant trials after applying the selection criteria. Discussion with a third reviewer (SL) was used to resolve doubt or disagreement about study inclusion. Inclusion criteria were as follows: 1) randomized controlled trials (RCTs) or quasi RCTs; 2) patients with one or multiple strokes that were confirmed by computed tomography or magnetic resonance imaging; 3) Ashworth Scale (AS) or Modified Ashworth Scale (MAS) of any joint ≥1; 4) comparison of any type of RC with or without CHM (oral CHM or topical CHM, such as steaming, compression, baths, and various external application therapies of CHM; CHM injection was not regarded as topical CHM and was not included in this study), or with placebo, with co-intervention being allowed as long as it was incorporated into all arms; and 5) studies that reported at least one of the following outcome measures: AS or MAS for spasticity severity as the primary outcome measure, Fugl-Meyer Assessment of Sensorimotor Recovery (FMA) for motor function and Barthel Index (BI) for assessment of activities of daily living as secondary outcome measures, and reporting of adverse events as a safety outcome. We excluded studies of patients with stroke symptoms caused by trauma, tumor, infection, and subdural hemorrhage or where the add-on effects of CHM could not be estimated due to the involvement of other interventions (i.e., CHM plus acupuncture plus rehabilitation therapies vs. rehabilitation therapies).

### Data Extraction

Two investigators (YC and CZ) independently extracted information on the characteristics of participants, study methods, and outcomes using a pre-designed form. A third reviewer (SL) checked all extracted data and corrected inconsistencies. If important data were unclear, unavailable, or suspected of duplication, authors of the trials were contacted *via* phone or emails for clarification.

### Quality Assessment (Risk of Bias)

Two researchers (YC and CZ) assessed the methodological quality of the included studies using the Cochrane risk-of-bias tool, following the Cochrane Handbook for Systematic Reviews of Interventions (version 5.1.0). Disagreement was resolved through discussion with a third investigator (SL) when necessary. Seven domains were assessed for each study: random sequence generation, allocation concealment, blinding of participants and personnel, blinding of outcome assessors, incomplete outcome data, selective reporting, and other bias.

### Data Synthesis and Analysis

#### Treatment Effects

Data synthesis was conducted using the Cochrane Review Manager software (RevMan 5.3). Mean difference (MD) and 95% confidence interval (CI) was used for continuous data, whereas the standard mean difference (SMD) was applied where the same outcome was reported using different scale ranges. Changes in AS or MAS, FMA, and BI was extracted or calculated for meta-analyses. Random-effect models were used for meta-analyses.

#### Subgroup Analysis and Sensitivity Analysis

The source of clinical heterogeneity among included trials was explored through subgroup analyses for baseline differences in terms of stroke onset (1 or >1 times), history of stroke (≤180 or >180 days), treatment duration (≤4 or >4 weeks), and preparation of herbal interventions. In consideration of the methodological quality, sensitivity analyses were performed based on the risk-of-bias judgements. In terms of herb analysis, *post hoc* subgroup analyses on the primary and secondary outcomes were conducted if sufficient data were available to explore the estimated effects of the individual or combination of the top five most frequently reported oral or topical herbs identified from this review.

### Publication Bias

Publication bias was assessed with a funnel plot and Egger’s linear regression test where more than 10 trials were included in a meta-analysis.

## Results

### Study Selection

**Figure 1:**
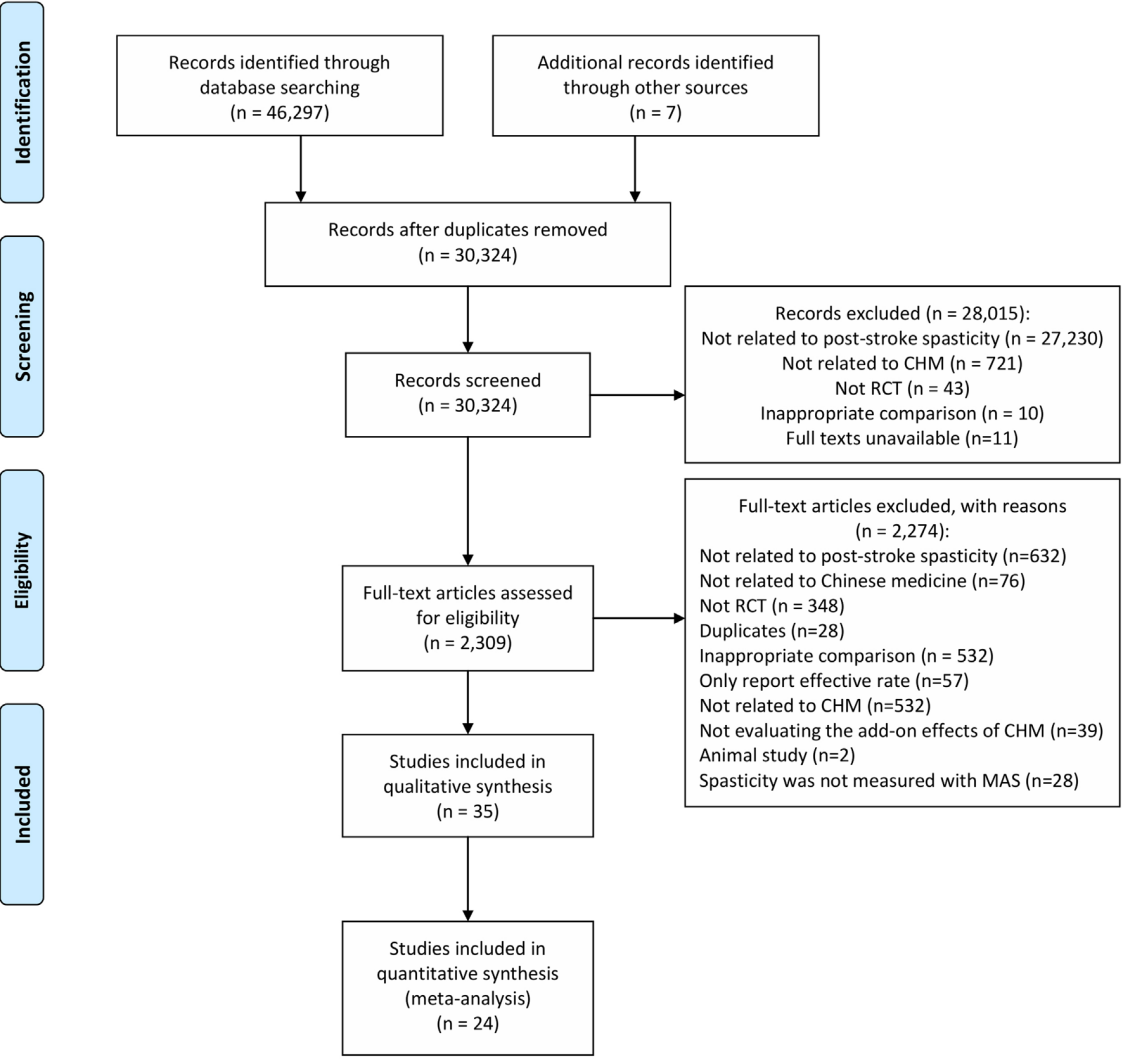
PRISMA flow chart. From: Moher D, Liberati A, Tetzlaff J, Altman DG, The PRISMA Group (2009). Preferred Reporting Items for Systematic Reviews and Meta-Analyses: The PRISMA Statement. PLoS Med 6(6):e1000097. doi: 10.1371/journal.pmed1000097. For more information, visit www.prisma-statement.org.

Using the comprehensive search, 46,304 studies were identified (**Figure 1**). A total of 2,309 possibly relevant studies were obtained for full-text screening. Thirty-five RCTs meeting our criteria were included in the systematic review, of which 24 were included in meta-analyses. The results of five RCTs (Zhang et al., 2008; Zhao, 2010; Huang et al., 2011; Chen, 2013; Zhao, 2013) could not be synthesized into meta-analyses because their reported data were incorrect or could not be pooled. Six studies (Zhu et al., 2002; Zhu et al., 2007; Zhang, 2009; Xie et al., 2011; Zhu et al., 2013; Weng, 2014) evaluated oral plus topical CHM; their results were not pooled for meta-analysis due to the diversity of interventions.

### Characteristics of Included Studies

All included RCTs were conducted in China and were published between 2002 and 2016 (**Table 1**). The study sample size ranged from 29 to 120. A total of 2,457 stroke patients with an average age of 61.72 years were included in these studies.

**Table 1:** Summary of basic characteristics.

Intervention	Author, year	Sample size (I/C)	Age (years)	Gender (% male)	Stroke type	First onset of stroke	Time from stroke onset	Spasticity severity	Outcome measures
Oral CHM	Cai, 2016	47/46	65.9	65.6	Cerebral infarction and hemorrhage	N/A	≤3 months	MAS > 0	MAS, CSS, ER
Oral CHM	Chen et al., 2014	25/25	59.1	68	Cerebral infarction	N/A	10–42 days	AS ≥ 2	AS, FMA, BI
Oral CHM	Chen, 2013	30/30	64.6	54.9	Cerebral infarction and hemorrhage	Yes	≤3 months	MAS: 1−3	CSI^a^, FMA^a^, BI^a^, EMG, TCM syndrome score
Oral CHM	He, 2016	50/50	60.7	61	N/A	N/A	4–87 days	MAS > 0	FMA, BI, ER
Oral CHM	Huang et al., 2011	20/15	68.6	57.1	N/A	N/A	≥3 months	MAS: 2−3	FMA^a^, wrist and ankle ROM
Oral CHM	Li and Wang, 2011	40/40	N/A	65	Cerebral infarction	N/A	2–82 days	MAS > 0	FMA, BI, ER
Oral CHM	Li et al., 2015	40/40	N/A	66.3	Cerebral infarction	N/A	≤90 days	MAS > 0	FMA, BI, ER
Oral CHM	Liu et al., 2014a	34/34	67.6	55.9	Cerebral infarction and hemorrhage	N/A	18–90 days	MAS ≥ 1+	MAS, BI
Oral CHM	Murat, 2016	36/36	61.5	62.5	Cerebral infarction and hemorrhage	Yes	2–5 weeks	MAS ≥ 1	BI, ER, TCM syndrome score
Oral CHM	Wang, 2013	35/34	N/A	60.9	Cerebral infarction and hemorrhage	N/A	30–180 days	MAS ≥ 1	MAS, FMA
Oral CHM	Wei et al., 2011	30/30	56.2	65.0	Cerebral infarction and hemorrhage	N/A	2 weeks–6 months	MAS > 0	MAS, FMA, ER
Oral CHM	Zhang et al., 2008	35/31	65.8	72.7	Cerebral infarction and hemorrhage	N/A	2 weeks–6 months	MAS: 1−3	MAS^a^, IEMG
Oral CHM	Zhang et al., 2012	60/40	64.14	68.0	Cerebral infarction and hemorrhage	Yes	14–180 days	MAS: 1−3	MAS, ER, VAS, Swelling score (upper limb), Berg balance score
Oral CHM	Zhao, 2013	30/30	63.5	65	Cerebral infarction	N/A	N/A	MAS ≥ 1	BI^a^, ER, AEs, neurological deficit score, Brunnstrom, motor patterns, TCM syndrome score
Oral CHM	Zhong, 2016	41/41	62.56	56.1	Cerebral infarction and hemorrhage	N/A	3–9 days	MAS ≥ 1	BI, ER
Topical CHM	Cao and Han, 2015	32/32	57.7	61.3	Cerebral infarction and hemorrhage	N/A	<365 days	MAS ≥ 1+	MAS, CSS, BI
Topical CHM	Chen et al., 2010	25/25	60	54	Cerebral infarction	N/A	≤6 months	MAS: 1-3	AS, AEs
Topical CHM	Ding, 2016	59/50	57.1	56.0	Cerebral infarction and hemorrhage	Yes	10–100 days	MAS ≥ 2	MAS, ROM, ER
Topical CHM	Huang, 2011	45/45	60.8	61.1	Cerebral infarction and hemorrhage	N/A	N/A	MAS ≥ 2	MAS, BI, AEs
Topical CHM	Jia et al., 2012	44/42	65.5	61.6	Cerebral infarction and hemorrhage	N/A	6–20 days	MAS ≥ 1+	MAS, FMA, BI, ER, VAS
Topical CHM	Lai, 2016	30/30	68.5	59.3	Cerebral infarction	N/A	N/A	MAS ≥ 1	MAS, CSS, FMA, BI, ER, AEs
Topical CHM	Li et al., 2008	30/30	57.2	58.3	Cerebral infarction and hemorrhage	N/A	N/A	MAS ≥ 1+	MAS, ER
Topical CHM	Ou et al., 2007	15/14	N/A	N/A	Cerebral infarction and hemorrhage	Yes	<1 year	MAS ≥ 2	MAS, step, walking speed
Topical CHM	Ou et al., 2014	20/21	59.4	53.7	Cerebral infarction and hemorrhage	Yes	<1 year	MAS ≥ 2	MAS, FMA, FIM, AEs
Topical CHM	Shen et al., 2007	31/30	60	55.7	Cerebral infarction	N/A	≤6 months	MAS: 1−3	AS, AEs
Topical CHM	Wang et al., 2014	24/24	57	62.5	Cerebral infarction and hemorrhage	N/A	30–151 days	MAS > 0	MAS, BI
Topical CHM	Zhang, 2016	60/60	62.1	60.0	Cerebral infarction and hemorrhage	Yes	17–180 days	MAS ≥ 1	MAS, BI
Topical CHM	Zhang et al., 2007	30/30	66.0	65.0	N/A	N/A	2–12 weeks	MAS: 1−3	FMA, FIM, ER
Topical CHM	Zhao, 2010	28/27	N/A	63.6	Cerebral infarction and hemorrhage	N/A	N/A	MAS ≥ 2	MAS^a^
Oral plus topical CHM	Weng, 2014	35/38	55.42	47.95	Cerebral infarction and hemorrhage	Yes	N/A	MAS: 1−3	MAS, FMA, BI, AEs
Oral plus topical CHM	Xie et al., 2011	60/60	59.25	60.83	Cerebral infarction and hemorrhage	N/A	<60 days	MAS > 1	MAS, FMA, BI
Oral plus topical CHM	Zhang, 2009	38/36	54.49	56.76	Cerebral infarction	N/A	N/A	AS > 0	FMA, BI, ER, TCM syndrome score, AEs
Oral plus topical CHM	Zhu et al., 2002	30/30	63.5	71.67	Cerebral infarction and hemorrhage	N/A	1–2 months	MAS ≥ 2	MAS, ER, AEs
Oral plus topical CHM	Zhu et al., 2007	31/31	63.1	59.68	Cerebral infarction and hemorrhage	N/A	1–2 months	MAS: 2−4	AS, BI, ER, AEs
Oral plus topical CHM	Zhu et al., 2013	30/30	64.35	51.67	N/A	N/A	N/A	AS: 1−3	AS, FMA, sEMG

AEs, adverse events; BI, Barthel Index; CHM, Chinese herbal medicine; CSS, Composite Spasticity Scale; EMG, electromyography; ER, effective rate; FIM, Functional Independent Measure; FMA, Fugl-Meyer Assessment of Sensorimotor Recovery; MAS, Modified Ashworth Scale; ROM, range of motion; sEMG, surface electromyography; TCM, traditional Chinese medicine; VAS, Visual Analogue Scale.

a reported data were incorrect or unable to be merged.

Fifteen studies (Zhang et al., 2008; Huang et al., 2011; Li and Wang, 2011; Wei et al., 2011; Zhang et al., 2012; Chen, 2013; Wang, 2013; Zhao, 2013; Chen et al., 2014; Liu et al., 2014a; Li et al., 2015; Cai, 2016; He, 2016; Murat, 2016; Zhong, 2016) investigated the add-on effects of oral CHM, 14 studies were on topical CHM (Ou et al., 2007; Shen et al., 2007; Zhang et al., 2007; Li et al., 2008; Chen et al., 2010; Zhao, 2010; Huang, 2011; Jia et al., 2012; Ou et al., 2014; Wang et al., 2014; Cao and Han, 2015; Ding, 2016; Lai, 2016; Zhang, 2016), and six studies were of oral plus topical CHM (Zhu et al., 2002; Zhu et al., 2007; Zhang, 2009; Xie et al., 2011; Zhu et al., 2013; Weng, 2014). The stroke history of the included patients was reported from 1 day to 1 year. Eight studies (Ou et al., 2007; Zhang et al., 2012; Chen, 2013; Ou et al., 2014; Weng, 2014; Ding, 2016; Murat, 2016; Zhang, 2016) only enrolled participants with a first-ever stroke. Nine trials (Zhu et al., 2002; Ou et al., 2007; Zhu et al., 2007; Zhao, 2010; Huang, 2011; Huang et al., 2011; Chen et al., 2014; Ou et al., 2014; Ding, 2016) merely included stroke patients whose AS or MAS were ≥2 at baseline. Reported CHM formulas varied greatly among the included studies, with a treatment duration ranging from 20 days to 3 months (**Table 2**). A variety of rehabilitation therapies were used as co-interventions (**Table 3**). Placebo was not used in any of the included trials. Twenty-three studies reported data on AS or MAS, 17 reported BI, and 14 reported FMA data (**Table 1**).

**Table 2:** Summary of intervention treatment.

Author, year	Oral CHM				Topical CHM			Formula	Ingredients^a^
	Preparation	Dosage	Frequency	Period	Duration	Frequency	Period		
Cai, 2016	Decoction	200 ml	bid	12 weeks	N/A	N/A	N/A	(oral) *Gua Lou Gui Zhi Tang*	*Gua Lou Gen, GuiZhi, Bai Shao, Sheng Jiang, Da Zao, Gan Cao*
Chen et al., 2014	Decoction	0.5 dose	bid	4 weeks	N/A	N/A	N/A	(oral) *Jia Wei Bu Yang Huan Wu Tang*	*Huang Qi, ChuanXiong, Dang Gui, Di Long, Tao Ren, Hong Hua, Jiang Can, Chi Shao, Tian Zhu Huang, Gua Lou*
Chen, 2013	Decoction	200 ml	bid	4 weeks	N/A	N/A	N/A	(oral) *Gua Lou GuiZhi Tang*	*Gua Lou Gen, GuiZhi, Bai Shao, Gan Cao, Sheng Jiang, Da Zao*
He, 2016	Decoction	1 dose	qd	28 days	N/A	N/A	N/A	(oral) *Tong Luo Jie Jing Tang*	*Dang Gui, Shu Di Huang, Niu Xi, Tian Ma, QuanXie, Chuan Shan Jia, Shen Jin Cao, Bai Shao, Sang Zhi, Ji XueTeng, Gou Teng, Wu Shao She, Di Long, Mu Gua*
Huang et al., 2011	Capsule	3g	tid	3 months	N/A	N/A	N/A	(oral) *Wen Jing Shu Jin Jiao Nang*	*Shu Di Huang, Lu Jiao Jiao, Ma Huang, Bai JieZi, Sheng Jiang, Rou Gui, Huang Qi, ChuanXiong, Dang Gui, Chi Shao, Tao Ren, Hong Hua, Ji XueTeng, Niu Xi, Di Long, Mu Gua, Shen Jin Cao, Bai Shao, Gan Cao*
Li and Wang, 2011	Decoction	1 dose	qd	28 days	N/A	N/A	N/A	(oral) *Tong Luo Jie Jing Tang*	*Dang Gui, Bai Shao, Shu Di Huang, Sang Zhi, Niu Xi, Ji XueTeng, Tian Ma, Gou Teng, QuanXie, Wu Shao She, Chuan Shan Jia, Di Long, Shen Jin Cao, Mu Gua*
Li et al., 2015	Decoction	1 dose	qd	28 days	N/A	N/A	N/A	(oral) *Yi Qi Rou Jin Tang*	*Huang Qi, Bai Zhu, Tao Ren, Hong Hua, Dang Gui, Chi Shao, ChuanXiong, Chang Pu, Dan Nan Xing, Niu Xi, Ji XueTeng, Shen Jin Cao, Sang Zhi, Ren Dong Teng, Di Long, Shan Zha*
Liu et al., 2014a	Decoction	0.5 dose	bid	2 months	N/A	N/A	N/A	(oral) Decoction without a name	*Huang Qi, Dang Gui, Bai Shao, Shan Zhu Yu, Sheng Di Huang, Shan Yao, Di Long, QuanXie, Gou Ji, Sang Ji Sheng*
Murat, 2016	Decoction	200 ml	bid	4 weeks	N/A	N/A	N/A	(oral) Decoction without a name	*Sheng Di Huang, Shu Di Huang, Bai Shao, Gan Cao, QuanXie, Wu Gong, Tao Ren, Hong Hua, Dan Shen, Di Long, TuBie Chong, Ji XueTeng, Gui Ban*
Wang, 2013	Capsule	N/A	tid	4 weeks	N/A	N/A	N/A	(oral) *Bu Chang Nao Xin Tong Jiao Nang*	*Huang Qi, Dang Gui, Chi Shao, ChuanXiong, Tao Ren, Hong Hua, Di Long, Mu Gua, Shen Jin Cao, Jiang Can, Wu Gong, QuanXie*
Wei et al., 2011	Decoction	100 ml	bid	4 weeks	N/A	N/A	N/A	(oral) *Rou Jin Tang*	*Sheng Di Huang, Bai Shao, Shan Zhu Yu, Shi Hu, Shen Jin Cao, Mu Gua, Lu Lu Tong, Sang Zhi, Gan Cao*
Zhang et al., 2008	Decoction	1 dose	qd	4 weeks	N/A	N/A	N/A	(oral) Decoction without a name	*Bai Shao, Sheng Di Huang, Gan Cao, Dang Gui, Ji XueTeng, Ru Xiang, Mo Yao, Di Long, Mu Gua, Shen Jin Cao*
Zhang et al., 2012	Decoction	0.5 dose	bid	3 weeks	N/A	N/A	N/A	(oral) *Shao Yao Gan Cao Tang*	*Bai Shao, Gan Cao, Shu Di Huang, Dang Gui*
Zhao, 2013	Decoction	250 ml	bid	4 weeks	N/A	N/A	N/A	(oral) *Zi Ni Wen Shen Yi Qi HuoXieTang*	*Fu Zi, Huang Qi, GuiZhi, Tao Ren, Hong Hua, Du Zhogn, Dang Shen, Xi Xin, ChuanXiong, Dang Gui, Di Long, Fu Ling, Bai Shao, Shen Jin Cao, Gan Cao*
Zhong, 2016	Decoction	0.5 dose	bid	4 weeks	N/A	N/A	N/A	(oral) *Shao Yao Gan Cao Tang*	*Bai Shao, Gan Cao*
Cao and Han, 2015	N/A	N/A	N/A	N/A	30 min	bid	30 days	(compression) Decoction without a name	*Jiang Can, Chi Shao, Shen Jin Cao, GuiZhi, Ge Gen, Mu Gua, Hong Hua, ZeXie, Fu Ling Pi, Di Long, Gan Cao*
Chen et al., 2010	N/A	N/A	N/A	N/A	30 min	qd	30 days	(steaming) Decoction without a name	*Bai Shao, Mu Gua, Shen Jin Cao, Dan Shen, GuiZhi, Dang Gui, ChuanXiong, Di Long, Gan Cao, Bing Pian*
Ding, 2016	N/A	N/A	N/A	N/A	30 min	qd	8 weeks	(compression) Decoction without a name	*Wu Tou, Cao Wu, Ji XueTeng, TouGu Cao, ChuanXiong, Su Mu, Shen Jin Cao, Hong Hua, GuiZhi, Ma Huang, Sang Zhi, You Song Jie, Dang Gui, Hua Jiao*
Huang, 2011	N/A	N/A	N/A	N/A	15–30 min	bid/tid	4 weeks	(steaming) *Shu Jin Tong Luo Fang*	*Bai Shao, Mu Gua, Ge Gen, Xi Xian Cao, Shen Jin Cao, TuBie Chong, ChuanXiong, XueJie, Hong Hua, Niu Xi, QuanXie, Wu Gong, Dan Shen, Sheng Di Huang, Dang Gui, TouGu Cao, Gan Cao*
Jia et al., 2012	N/A	N/A	N/A	N/A	40 min	qd	4 weeks	(steaming) Decoction without a name	*Hong Hua, Dan Shen, Dang Gui, ChuanXiong, Ji XueTeng, Mu Gua, Xi Xian Cao, Shen Jin Cao, Wei Ling Xian, QiangHuo, Du Huo, Sang Zhi, GuiZhi, Cang Zhu, Bai Zhu, Di Long, Bai Shao, Gan Cao*
Ou et al., 2007	N/A	N/A	N/A	N/A	20 min	qd	20 days	(steaming) *Shu JinHuo Luo Xi Ji*	*Huang Qi, Dang Gui, Dang Shen, Tao Ren, Hong Hua, ChuanXiong, Su Mu, Sang Zhi, Shen Jin Cao, Ji XueTeng, Mu Gua, Wei Ling Xian, Dan Shen, Ma Qian Zi*
Ou et al., 2014	N/A	N/A	tid	N/A	20 min	qd	20 days	(steaming) *Shu JinHuo Luo Xi Ji*	*Huang Qi, Dang Gui, Dang Shen, Tao Ren, Hong Hua, ChuanXiong, Su Mu, Sang Zhi, Shen Jin Cao, Ji XueTeng, Mu Gua, Wei Ling Xian, Dan Shen, Ma Qian Zi*
Shen et al., 2007	N/A	N/A	N/A	N/A	20–30 min	qd	30 days	(compression) Decoction without a name	*Dang Gui, ChuanXiong, Bing Pian, Niu Xi, TouGu Cao, Wei Ling Xian, Hong Hua, Fang Feng, Ai Ye, GuiZhi, Zhe Chong, Huang Jiu, Ci, Tong You*
Wang et al., 2014	N/A	N/A	N/A	N/A	20 min	qd	28 days	(compression) Decoction without a name	*Shen Jin Cao, Bai Shao, Gan Cao, Dang Gui, Di Long, Mai Dong, Fang Feng, Wu Gong*
Zhang, 2016	N/A	N/A	N/A	N/A	60 min	6 times/week	4 weeks	(steaming) *Jie Jing Shu Jin Tang*	*Tian Ma, Gou Teng, Wu Gong, Fang Feng, Shen Jin Cao, Ji XueTeng, Wei Ling Xian, Bai Shao, Mai Dong, Dang Gui, Tao Ren, Hong Hua*
Zhang et al., 2007	N/A	N/A	N/A	N/A	20 min	qd	4 weeks	(steaming) Decoction without a name	*Bai Shao, Wang Jiang Nan, Shen Jin Cao, Mu Gua, Sang Zhi, GuiZhi, Hong Hua, Dang Gui, Ru Xiang, Mo Yao*
Zhao, 2010	N/A	N/A	N/A	N/A	40 min	qd	28 days	(foot bath) Decoction without a name	*Hong Hua, Tao Ren, Dang Gui, Dan Shen, Mu Gua*
Weng, 2014	Decoction	100 ml	bid	4 weeks	N/A	bid	4 weeks	(oral+compression) *Jie Jing He Ji*	*Bai Shao, Gan Cao, Wang Jiang Nan, Mu Gua, Ru Xiang, Mo Yao, QuanXie, Dan Shen, Huang Jiu*
Xie et al., 2011	Decoction	150 ml	qd	28 days	20 min	qd	28 days	(bath) *Rou Jin Tang*	*Huang Qi, Dang Gui, Ji XueTeng, Shan Zhu Yu, Wu Gong*
								(compression) Decoction without a name	*Bing Pian, Tan Xiang, Dang Gui, Bai Shao, Wu Mei*
Zhang, 2009	Decoction	150 ml	bid	28 days	15–30 min	bid	28 days	(oral) Decoction without a name	*Bai Shao, Gan Cao, Shen Jin Cao,* 川*Mu Gua, Ji XueTeng, Chuan Shan Jia, Niu Xi, Sang Zhi, Tian Ma, Jiang Can*
								(topical) Decoction without a name	*Wu Tou, Fang Feng, GuiZhi, Hua Jiao, Hong Hua, Dang Gui, Huang Qi, TouGu Cao, Shen Jin Cao, Wei Ling Xian*
Zhu et al., 2002	CHM syrup	10 ml	tid	30 days	N/A	qid	30 days	(oral+compression) *Jie Jing He Ji*	*Bai Shao, Gan Cao, Wang Jiang Nan, Mu Gua, QuanXie, Dan Shen, Huang Jiu*
Zhu et al., 2007	CHM syrup	10 ml	tid	30 days	N/A	qid	30 days	(oral+compression) *Jie Jing He Ji*	*Bai Shao, Gan Cao, Wang Jiang Nan, Mu Gua, Ru Xiang, Mo Yao, QuanXie, Dan Shen, Huang Jiu*
Zhu et al., 2013	CHM syrup	10 ml	tid	30 days	N/A	qid	30 days	(oral+compression) *Shao Yao Gan Cao Tang*	*Bai Shao, Gan Cao*

bid, twice per day; CHM, Chinese herbal medicine; min, minutes; N/A, not available; qd, once per day; qid, four times per day; tid, three times per day.

a The ingredients of formulas were presented with Chinese pinyin. Correspondent scientific names were available in the book “Dan Bensky. Editor. Chinese Herbal Medicine: Materia Medica. Third Edition. WA: Eastland Press. Inc; 2004”.

**Table 3:** Summary of rehabilitation treatment.

Study ID	Control method	Pharmacotherapy			Rehabilitation therapy		
		Dose	Frequency	Treatment period	Duration in each treatment section	Frequency (times/day × times/week)	Treatment period
Cai, 2016	Rehabilitation programs and Baclofen	Increased from 10 mg to 75 mg	qd	12 weeks	45 min	1 × 7	12 weeks
Cao and Han, 2015	Rehabilitation programs	N/A	N/A	N/A	N/A	N/A	30 days
Chen et al., 2010	Baclofen	Increased from 5 mg to 10 mg	tid	30 days	N/A	N/A	30 days
Chen et al., 2014	Rehabilitation programs	N/A	N/A	N/A	60 min	1 × 6	4 weeks
Chen, 2013 (B)	Rehabilitation programs	N/A	N/A	N/A	N/A	1 × 6	4 weeks
Ding, 2016	Rehabilitation programs	N/A	N/A	N/A	40 min	1 × 7	8 weeks
He, 2016	Rehabilitation programs	N/A	N/A	N/A	40 min	1 × 6	28 days
Huang, 2011	Rehabilitation programs	N/A	N/A	N/A	60 min	1 × 5	4 weeks
Huang et al., 2011	Rehabilitation programs	N/A	N/A	N/A	N/A	N/A	3 months
Jia et al., 2012	Rehabilitation programs	N/A	N/A	N/A	45 min	1 × 6	4 weeks
Lai, 2016	Rehabilitation programs	N/A	N/A	N/A	N/A	1 × 7	30 days
Li et al., 2008	Rehabilitation programs	N/A	N/A	N/A	N/A	N/A	6 weeks
Li and Wang, 2011	Rehabilitation programs	N/A	N/A	N/A	40 min	1 × 6	28 days
Li et al., 2015	Rehabilitation programs	N/A	N/A	N/A	N/A	N/A	3 months
Liu et al., 2014a	Rehabilitation programs	N/A	N/A	N/A	30 min	1 × 7	2 months
Murat, 2016	Rehabilitation programs	N/A	N/A	N/A	N/A	N/A	4 weeks
Ou et al., 2007	Botulinum toxin and rehabilitation programs	100–300 units in total	N/A	N/A	30–60 min	(1 to 2) × 7	2 months
Ou et al., 2014	Botulinum toxin and rehabilitation programs	20–40 units/injection point	N/A	N/A	N/A	N/A	2 months
Shen et al., 2007	Rehabilitation programs	N/A	N/A	N/A	N/A	N/A	30 days
Wang et al., 2014	Rehabilitation programs	N/A	N/A	N/A	45 min	1 × 6	28 days
Wang, 2013	Rehabilitation programs	N/A	N/A	N/A	N/A	N/A × 6	4 weeks
Wei et al., 2011	Rehabilitation programs	N/A	N/A	N/A	1 h	2 × 7	4 weeks
Weng, 2014	Tizanidine and rehabilitation programs	2–4 mg	tid	4 weeks	45 min	1 × 5	4 weeks
Xie et al., 2011	Rehabilitation programs	N/A	N/A	N/A	90 min	2 × 7	28 days
Zhang, 2016	Rehabilitation programs	N/A	N/A	N/A	N/A	1 × 5	4 weeks
Zhang, 2009	Rehabilitation programs	N/A	N/A	N/A	45–60 min	(1 to 2) × 6	28 days
Zhang et al., 2007	Rehabilitation programs	N/A	N/A	N/A	45 min	1 × 6	4 weeks
Zhang et al., 2008	Rehabilitation programs	N/A	N/A	N/A	45 min	1 × 6	4 weeks
Zhang et al., 2012	Rehabilitation programs	N/A	N/A	N/A	45 min	1 × 6	3 weeks
Zhao, 2013	Rehabilitation programs	N/A	N/A	N/A	N/A	N/A	4 weeks
Zhao, 2010	Rehabilitation programs	N/A	N/A	N/A	40 min	1 × 7	28 days
Zhong, 2016	Rehabilitation programs	N/A	N/A	N/A	30 min	1 × 7	4 weeks
Zhu et al., 2002	Rehabilitation programs	N/A	N/A	N/A	N/A	N/A	30 days
Zhu et al., 2007	Rehabilitation programs	N/A	N/A	N/A	N/A	N/A	30 days
Zhu et al., 2013	Rehabilitation programs	N/A	N/A	N/A	30 min	1 × 6	4 weeks

bid, twice per day; N/A, not available; qd, once per day; tid, three times per day.

### Risk of Bias of Included Studies

**Figure 2:**
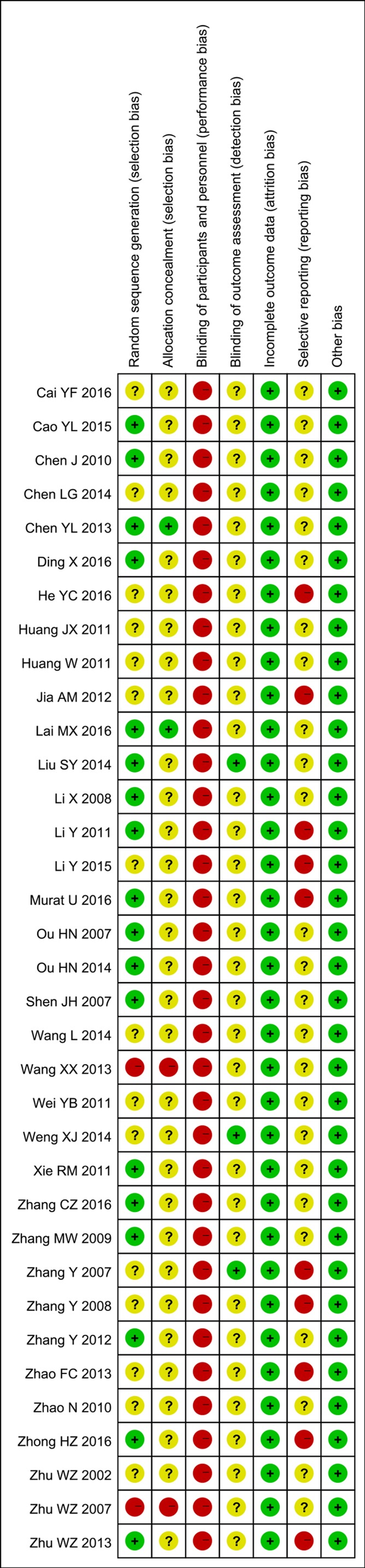
Risk-of-bias summary.

Eighteen studies (Ou et al., 2007; Shen et al., 2007; Li et al., 2008; Zhang, 2009; Chen et al., 2010; Li and Wang, 2011; Xie et al., 2011; Zhang et al., 2012; Chen, 2013; Zhu et al., 2013; Liu et al., 2014a; Ou et al., 2014; Cao and Han, 2015; Ding, 2016; Lai, 2016; Murat, 2016; Zhang, 2016; Zhong, 2016) were assessed as low risk of bias in random sequence generation with adequate methods; two trials (Zhu et al., 2007; Wang, 2013) were assessed as high risk of bias because they allocated patients based on the date of admission; others were of unclear risk due to a lack of information. Allocation was well concealed in only two studies (Chen, 2013; Lai, 2016), whereas another two (Zhu et al., 2007; Wang, 2013) were assessed as high risk of bias because participants were allocated based on their case record number. Blinding of participants and personnel was attempted in none of the included trials, but three trials (Zhang et al., 2007; Liu et al., 2014a; Weng, 2014) performed blinding in outcome assessors. All included trials were assessed as low risk of bias in incomplete outcome data. None of the included studies had prospectively registered protocols, and 10 studies (Zhang et al., 2007; Zhang et al., 2008; Li and Wang, 2011; Jia et al., 2012; Zhao, 2013; Zhu et al., 2013; Li et al., 2015; He, 2016; Murat, 2016; Zhong, 2016) did not report the results of all pre-defined outcomes mentioned in the Methods sections. Risk-of-bias assessment is summarized in **Figure 2**.

### Synthesis of Results

Results of meta-analyses are presented below for oral CHM and topical CHM, separately (**Table 4**).

**Table 4:** Summary of meta-analyses results.

	Outcome measure	No. of studies	Effects	I^2^ (%)
Oral CHM	Upper-limb AS/MAS	3	SMD −1.79, 95% CI: −3.00 to −0.57*	94
	Lower-limb AS/MAS	3	SMD −1.01, 95% CI: −1.43, −0.59*	55
	Overall motor FMA	3	MD 12.14, 95% CI: 1.57, 22.71*	89
	Upper-limb motor FMA	3	MD 7.64, 95% CI: −1.29, 16.57	97
	Lower-limb motor FMA	2	MD 4.03, 95% CI: 1.90, 6.17*	61
	BI	7	MD 13.15, 95% CI: 4.37, 21.93*	98
Topical CHM	Upper-limb AS/MAS	8	SMD −1.06, 95% CI: −1.40, −0.72*	72
	Lower-limb AS/MAS	5	SMD −1.16, 95% CI: −1.83, −0.49*	84
	Overall motor FMA	2	MD 5.56, 95% CI: 2.38, 8.74*	0
	Upper-limb motor FMA	2	MD 5.88, 95% CI: 4.09, 7.68*	0
	BI	6	MD 12.01, 95% CI: 2.81, 21.22*	99

*Significant add-on effect was detected by meta-analysis.

#### Add-On of Oral CHM to RC

**Figure 3:**
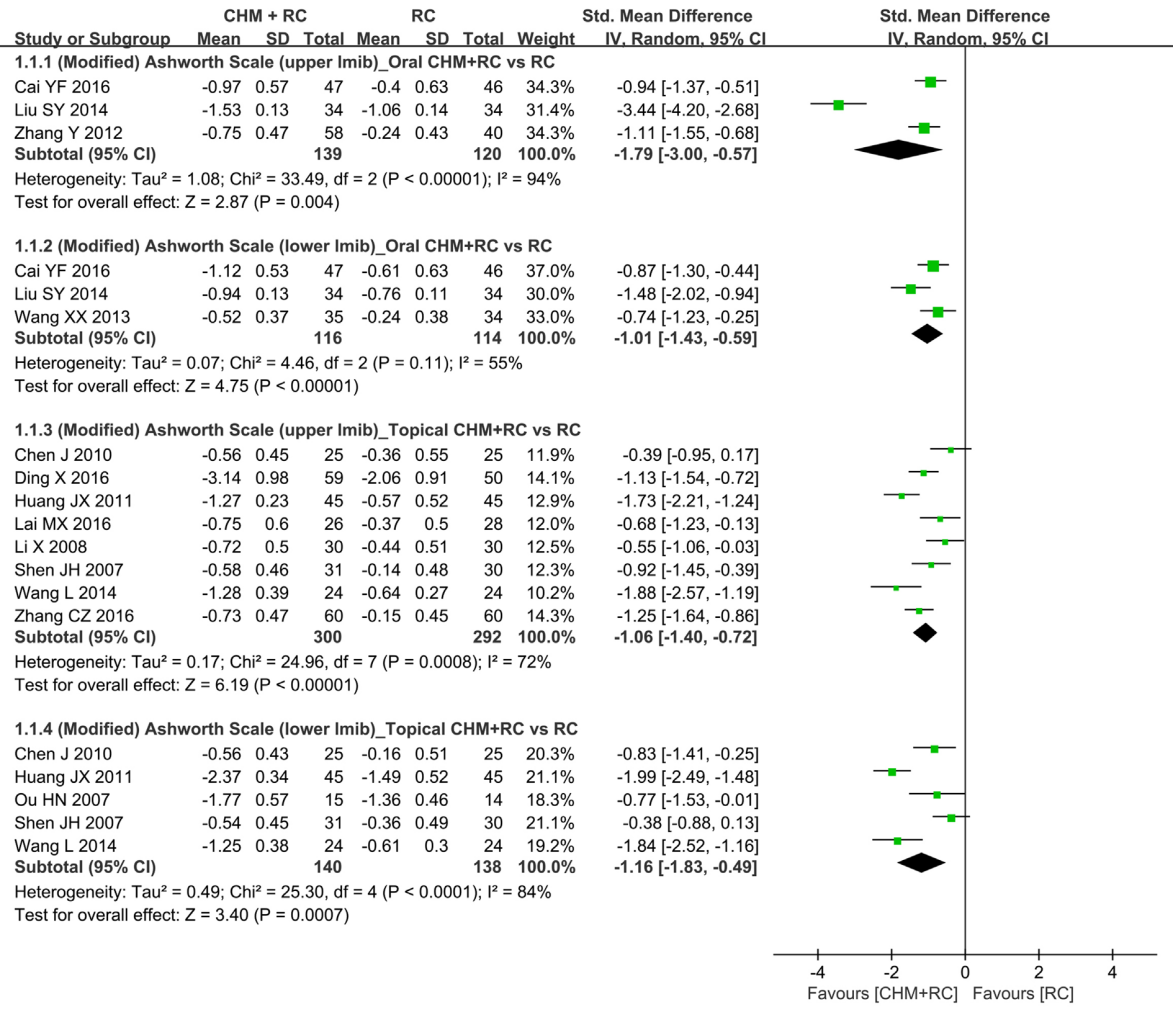
Forest plot of (Modified) Ashworth Scale.

Significant add-on effects of oral CHM were found in terms of changes in scores of AS or MAS of the upper limbs (three studies: Zhang et al., 2012; Liu et al., 2014a; Cai, 2016; SMD −1.79, 95% CI: −3.00 to −0.57, *I* ^2^ = 94%) and lower limbs (three studies: Wang, 2013; Liu et al., 2014a; Cai, 2016; SMD −1.01, 95% CI: −1.43 to −0.59, *I* ^2^ = 55%), although with moderate to high heterogeneity (**Table 4** and **Figure 3**).

**Figure 4:**
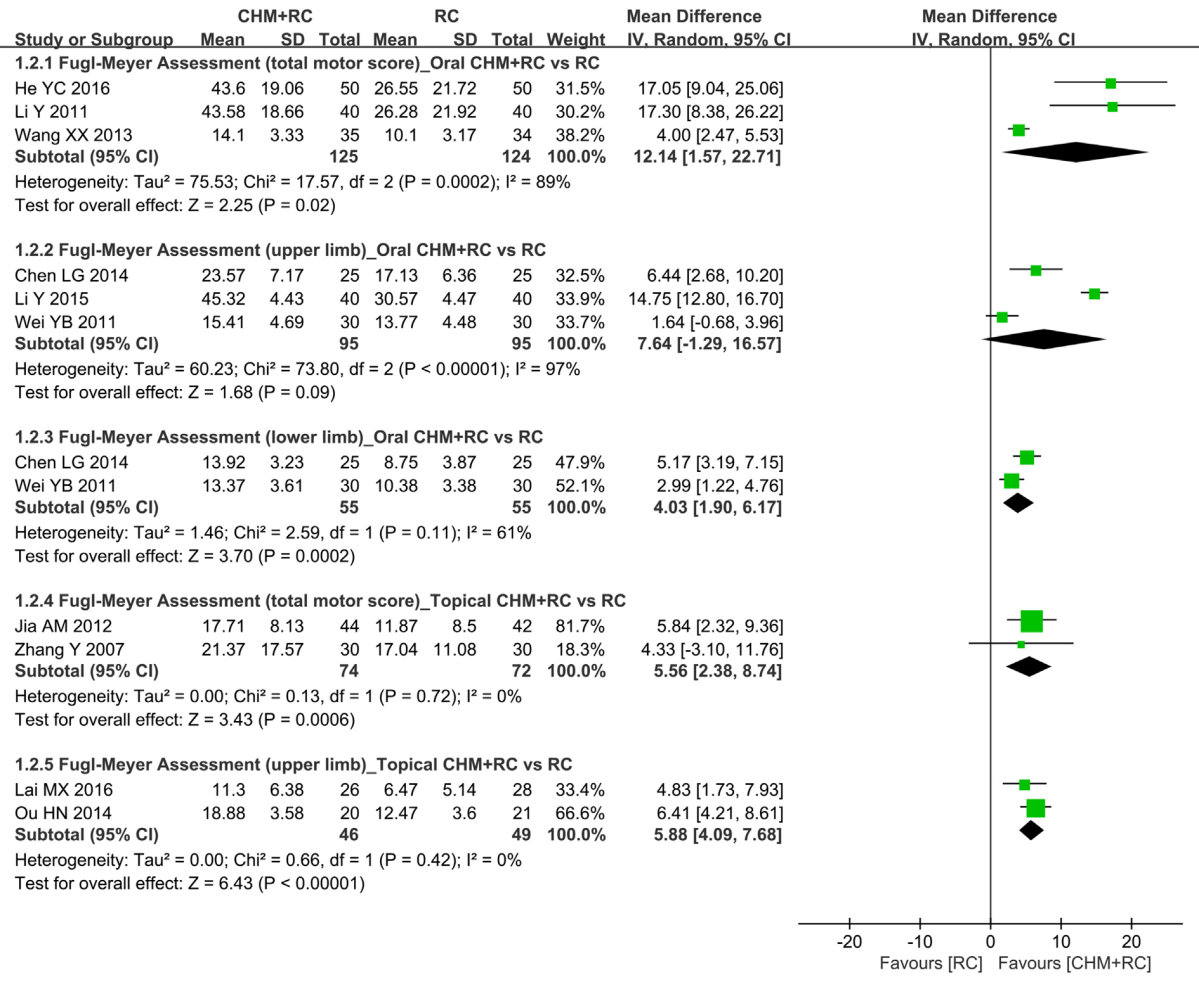
Forest plot of Fugl-Meyer Assessment.

In terms of the improvement of overall motor function measured using the FMA, combining oral CHM and RC was estimated to be significantly superior to RC alone (three studies: Li and Wang, 2011; Wang, 2013; He, 2016; MD 12.14, 95% CI: 1.57 to 22.71, *I* ^2^ = 89%) (**Table 4** and **Figure 4**). Similarly, benefits of adding oral CHM to RC were seen in the FMA score changes for the lower extremities (two studies: Wei et al., 2011; Chen et al., 2014; MD 4.03, 95% CI:1.90 to 6.17, *I* ^2^ = 61%), but not in that of the upper limbs (three studies: Wei et al., 2011; Chen et al., 2014; Li et al., 2015; MD 7.64, 95% CI: −1.29 to 16.57, *I* ^2^ = 97%).

**Figure 5:**
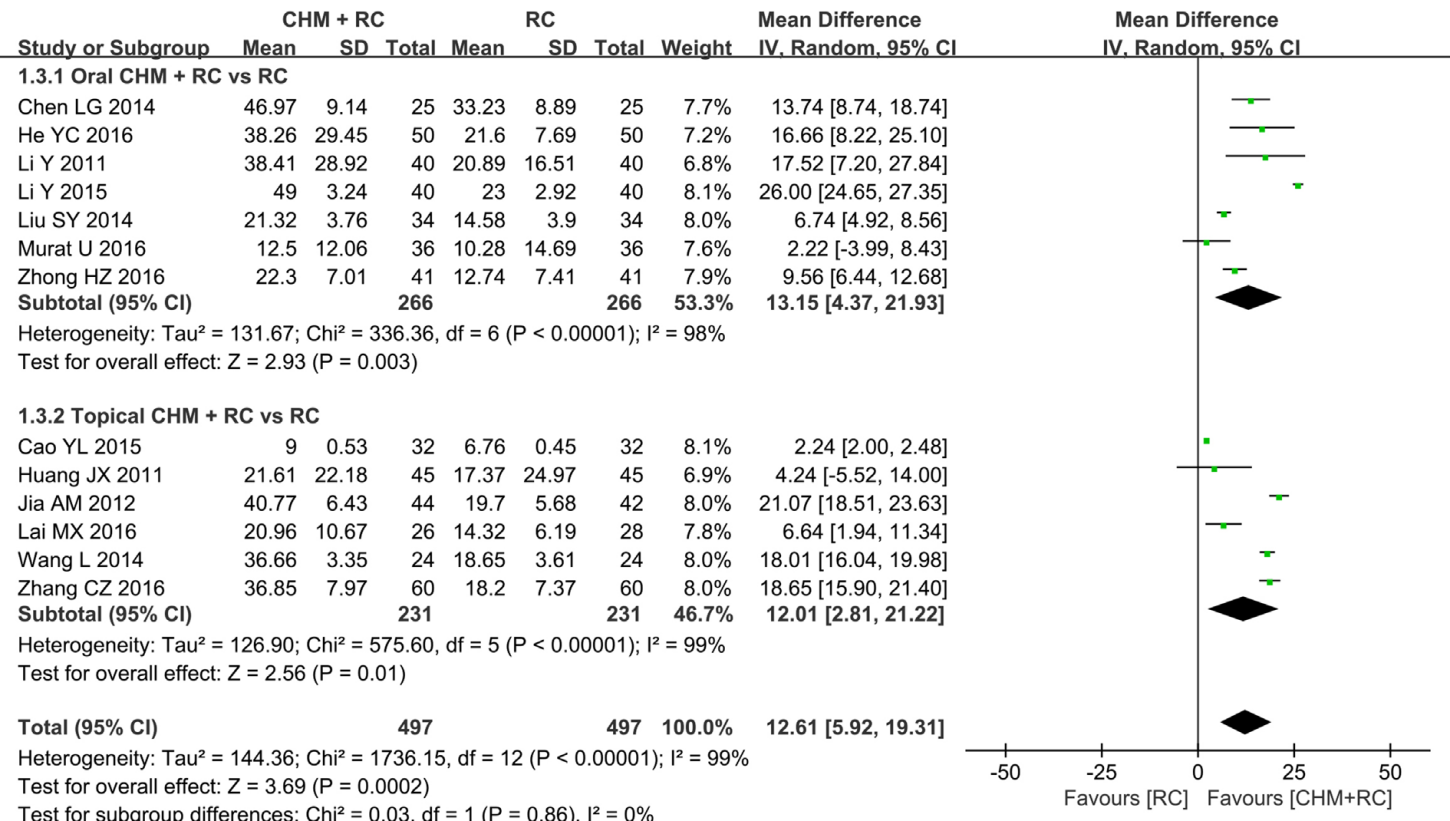
Forest plot of Barthel Index.

Seven included trials (Li and Wang, 2011; Chen et al., 2014; Liu et al., 2014; Li et al., 2015; He, 2016; Murat, 2016; Zhong, 2016) reported changes to the BI results and were pooled for meta-analysis. Results showed that the combination of oral CHM and RC yielded more improvement in the BI than RC alone (MD 13.15, 95% CI: 4.37 to 21.93), although with high heterogeneity (*I* ^2^ = 98%) (**Table 4** and **Figure 5**).

#### Add-On of Topical CHM to RC

Compared to RC alone, adding topical CHM further decreased AS or MAS in the upper limbs (eight studies: Shen et al., 2007; Li et al., 2008; Chen et al., 2010; Huang, 2011; Wang et al., 2014; Ding, 2016; Lai, 2016; Zhang, 2016; SMD −1.06, 95% CI: −1.40 to −0.72, *I* ^2^ = 72%) and lower limbs (five studies: Ou et al., 2007; Shen et al., 2007; Chen et al., 2010; Huang, 2011; Wang et al., 2014; SMD −1.16, 95% CI: −1.83 to −0.49, *I* ^2^ = 84%) (**Table 4**), although with high heterogeneity detected in both analyses.

Synthesis of FMA (total motor function) changes from two trials (Zhang et al., 2007; Jia et al., 2012) showed superior effects of topical CHM combined with RC compared to RC alone (MD 5.56, 95% CI: 2.38 to 8.74, *I* ^2^ = 0%) (**Table 4**). Similarly, meta-analysis results of another two studies (Ou et al., 2014; Lai, 2016) showed greater improvement in FMA (upper-limb motor function) with topical CHM added to RC (MD 5.88, 95% CI: 4.09 to 7.68, *I* ^2^ = 0%) than with RC alone (**Table 4**).

Compared to RC alone, a combination of topical CHM and RC further improved BI results, as shown in a meta-analysis of six studies (Huang, 2011; Jia et al., 2012; Wang et al., 2014; Cao and Han, 2015; Lai, 2016; Zhang, 2016) (MD 12.01, 95% CI: 2.81 to 21.22, *I* ^2^ = 99%) (**Table 4**).

### Safety Assessment

In total, 10 of the included studies addressed the safety of CHM; the remaining 25 studies did not provide information on adverse events. One study (Lai, 2016) reported one case of skin allergy in the intervention group receiving topical CHM. Although the symptom was evaluated as mild by the physician and was alleviated after 3 days, the patient dropped out of the study due to this event, without further confirmation of causality. Another study reported one patient in the treatment group of topical CHM who experienced transient influenza-like symptoms after Botox injection (Ou et al., 2014), which was considered not related to the use of CHM.

### Subgroup Analyses and Sensitivity Analyses

#### Add-On of Oral CHM to RC

Due to the limited number of included studies, there were insufficient data for subgroup analysis. In terms of sensitivity analysis of BI synthesis results, when only studies with low risk of bias in sequence generation were included, significant results remained and heterogeneity reduced to 66% (four studies: Li and Wang, 2011; Liu et al., 2014a; Murat, 2016; Zhong, 2016; MD 7.81, 95% CI: 4.31 to 11.31) (**Table 5**).

**Table 5:** Sensitivity analysis.

	Oral CHM add-on to RC	Risk of bias	No. of studies	Effects	I2 (%)
Oral CHM	Upper-limb AS or MAS		3	SMD –1.79, 95% CI: –3.00 to -0.57	94
	Sequence generation	Low	N/A	N/A	N/A
	Blinding of assessors	Low	1	SMD –3.44, 95% CI: −4.20 to -2.68	/
	Lower-limb AS or MAS		3	SMD –1.01, 95% CI: −1.43 to -0.59	55
	Sequence generation	Low	1	SMD –1.48, 95% CI: −2.02 to -0.94	/
	Blinding of assessors	Low	1	SMD –1.48, 95% CI: −2.02 to -0.94	/
	Overall motor FMA		3	MD 12.14, 95% CI: 1.57 to 22.71	89
	Sequence generation	Low	1	MD 17.30, 95% CI: 8.38 to 26.22	/
	Blinding of assessors	Low	N/A	N/A	N/A
	BI		7	MD 13.15, 95% CI: 4.37 to 21.93	98
	Sequence generation	Low	4	MD 7.81, 95% CI: 4.31 to 11.31*	66
	Blinding of assessors	Low	N/A	N/A	N/A
Topical CHM	Upper-limb AS or MAS		8	SMD -1.06, 95% CI: −1.40 to −0.72	72
	Sequence generation	Low	6	SMD -0.86, 95% CI: −1.14 to −0.58*	50
	Blinding of assessors	Low	N/A	N/A	N/A
	Lower-limb AS or MAS		5	SMD −1.16, 95% CI: −1.83 to −0.49	84
	Sequence generation	Low	3	SMD −0.61, 95% CI: −0.96 to −0.27*	0
	Blinding of assessors	Low	N/A	N/A	N/A
	BI		6	MD 12.01, 95% CI: 2.81 to 21.22	99
	Sequence generation	Low	3	MD 9.16, 95% CI: −2.37 to 20.69	99
	Blinding of assessors	Low	N/A	N/A	N/A

AS or MAS, Ashworth Scale or Modified Ashworth Scale; BI, Barthel Index; CHM, Chinese herbal medicine; FMA, Fugl-Meyer Assessment of Sensorimotor Recovery; N/A, not applicable.

*Heterogeneity reduced.

#### Add-On of Topical CHM to RC

For AS or MAS in the upper extremity, heterogeneity reduced to 0% in the subgroup where only patients with first-stroke onset were included. Due to the limited number of studies, subgroup analysis on FMA was not possible. With regard to BI, the subgroup where patients were within 180 days after stroke (three studies: Jia et al., 2012; Wang et al., 2014; Zhang, 2016; SMD 19.14, 95% CI: 17.29 to 20.98, *I* ^2^ = 43%) demonstrated a greater effect than observed in the subgroup of patients with a post-stroke period exceeding 180 days (three studies: Huang, 2011; Cao and Han, 2015; Lai, 2016; SMD 3.53, 95% CI: 0.51 to 6.54, *I* ^2^ = 43%) (**Table 6**). In terms of the administration of CHM, an add-on effect was detected when the CHM was used as steaming therapy for the outcomes of lower-limb AS or MAS (SMD −1.22, 95% CI: −2.06 to −0.39, *I* ^2^ = 82%) and BI (MD 17.12, 95% CI: 11.92 to 22.32, *I* ^2^ = 82%), while there was no add-on benefit for AS or MAS of lower limb (SMD −1.09, 95% CI: −2.52 to 0.34, *I* ^2^ = 91%) and BI (MD8.98, 95% CI: −2.81 to 20.76, *I* ^2^ = 99%) when CHM was used for compression.

**Table 6:** Subgroup analysis.

Analysis	Subgroups		Upper-limb AS or MAS^a^	Lower-limb AS or MAS^a^	Barthel Index^b^
Subgroup analysis	All studies		SMD −1.06, 95% CI: −1.40 to −0.72, *I* ^2 ^= 72%, 8(300/292)	SMD –1.16, 95% CI: –1.83 to –0.49, *I* ^2 ^= 84%, 5(140/138)	MD 12.01, 95% CI: 2.81, 21.22, *I* ^2 ^= 99%, 6(231/231)
	First onset of stroke	Yes	SMD −1.19, 95% CI: −1.48 to –0.91, *I* ^2 ^= 0%, 2(119/110)^d^	N/A	N/A
		Unspecified	SMD –1.01, 95% CI: –1.50 to –0.53, *I* ^2 ^= 79%, 6(181/182)	N/A	N/A
	Treatment duration	>4 weeks	N/A	N/A	N/A
		≤4 weeks	N/A	N/A	N/A
	Post-stroke period	≤180 days	SMD –1.10, 95% CI: –1.48 to –0.71, *I* ^2 ^= 67%, 5(199/189)	SMD –0.99, 95% CI: –1.80 to –0.18, *I* ^2^ = 82%, 3(80/79)	MD 19.14, 95% CI: 17.29 to 20.98, *I* ^2 ^= 43%, 3(128/126)^d^
		>180 days	SMD –0.99, 95% CI: –1.75 to –0.24, *I* ^2 ^= 84%, 3(101/103)	SMD –1.41, 95% CI: –2.60 to –0.22, *I* ^2 ^= 85%, 2(60/59)	MD 3.53, 95% CI: 0.51 to 6.54, *I* ^2 ^= 43%, 3(103/105)^d^
	Preparation	Compression	SMD –1.12, 95% CI: –1.54 to –0.69, *I* ^2 ^= 60%, 4(140/132)	SMD –1.09, 95% CI: –2.52 to 0.34, *I* ^2 ^= 91%, 2(55/54)^c^	MD 8.98, 95% CI: –2.81 to 20.76, *I* ^2 ^= 99%, 3(82/84)^c^
		Steaming therapy	SMD –0.99, 95% CI: –1.58 to –0.41, *I* ^2 ^= 83%, 4(160/160)	SMD –1.22, 95% CI: –2.06 to –0.39, *I* ^2 ^= 82%, 3(85/84)	MD 17.12, 95% CI: 11.92 to 22.32, *I* ^2 ^= 82%, 3(149/147)
*Post hoc* analysis with herbal ingredients	BS	Included	SMD -1.15, 95% CI: –1.69 to –0.61, *I* ^2 ^= 82%, 5(184/184)	SMD –1.55, 95% CI: –2.29 to –0.82, *I* ^2 ^= 79%, 3(94/94)	MD 18.01, 95% CI: 14.91 to 21.12, *I* ^2 ^= 75%, 4(173/171)
		Not included	SMD –0.96, 95% CI: –1.24 to –0.68, *I* ^2 ^= 0%, 3(116/108)^d^	SMD –0.50, 95% CI: –0.92 to –0.08, *I* ^2 ^= 0%, 2(44/44)^d^	MD 3.79, 95% CI: –0.33 to 7.91, *I* ^2 ^= 70%, 2(58/60)^c^
	DG	Included	SMD –1.21, 95% CI: –1.58 to –0.83, *I* ^2 ^= 71%, 4(244/234)	N/A	MD 18.01, 95% CI: 14.91 to 21.12, *I* ^2 ^= 75%, 4(173/171)
		Not included	SMD –0.61, 95% CI: –0.99 to –0.23, *I* ^2 ^= 0%, 2(56/58)^d^	N/A	MD 3.79, 95% CI: –0.33 to 7.91, *I* ^2 ^= 70%, 2(58/60)^c^
*Post hoc* analysis with herbal ingredients	BS+DG	Included	SMD –1.30, 95% CI: –1.90 to –0.71, *I* ^2 ^= 81%, 4(154/154)	SMD –1.55, 95% CI: –2.29 to –0.82, *I* ^2 ^= 79%, 3(94/94)	MD 18.01, 95% CI: 14.91 to 21.12, *I* ^2 ^= 75%, 4(173/171)
		Not included	SMD –0.86, 95% CI: –1.13 to –0.59, *I* ^2^ = 16%, 4(146/138)^d^	SMD –0.50, 95% CI: –0.92 to –0.08, *I* ^2 ^= 0%, 2(44/44)^d^	MD 3.79, 95% CI: –0.33 to 7.91, *I* ^2 ^= 70%, 2(58/60)^c^
	BS+SJC	Included	SMD –1.15, 95% CI: –1.69 to –0.61, *I* ^2 ^= 82%, 5(184/184)	SMD -1.55, 95% CI: –2.29 to –0.82, *I* ^2 ^= 79%, 3(94/94)	MD 18.01, 95% CI: 14.91 to 21.12, *I* ^2 ^= 75%, 4(173/171)
		Not included	SMD –0.96, 95% CI: –1.24 to –0.68, *I* ^2 ^= 0%, 3(116/108)^d^	SMD –0.50, 95% CI: –0.92 to –0.08, *I* ^2^ = 0%, 2(44/44)^d^	MD 3.79, 95% CI: –0.33 to 7.91, *I* ^2 ^= 70%, 2(58/60)^c^
	DG+SJC	Included	SMD –1.26, 95% CI: –1.70 to –0.82, *I* ^2 ^= 75%, 5(213/204)	N/A	MD 18.01, 95% CI: 14.91 to 21.12, *I* ^2^ = 75%, 4(173/171)
		Not included	SMD –0.72, 95% CI: –1.02 to –0.41, *I* ^2 ^= 0%, 3(87/88)^d^	N/A	MD 3.79, 95% CI: –0.33 to 7.91, *I* ^2 ^= 70%, 2(58/60)^c^
	BS+DG+HH	Included	SMD –1.56, 95% CI: –1.94 to –1.17, *I* ^2 ^= 43%, 3(129/129)^d^	SMD –1.93, 95% CI: –2.34 to –1.53, *I* ^2 ^= 0%, 2(69/69)^d^	MD 18.01, 95% CI: 14.91 to 21.12, *I* ^2 ^= 75%, 4(173/171)
		Not included	SMD –0.77, 95% CI: –1.04 to –0.49, *I* ^2 ^= 32%, 5(171/163)^d^	SMD –0.61, 95% CI: –0.96 to –0.27, *I* ^2 ^= 0%, 3(71/69)^d^	MD 3.79, 95% CI: –0.33 to 7.91, *I* ^2 ^= 70%, 2(58/60)^c^
	BS+DG+SJC	Included	SMD –1.30, 95% CI: –1.90 to –0.71, *I* ^2 ^= 81%, 4(154/154)	SMD –1.55, 95% CI: –2.29 to –0.82, *I* ^2 ^= 79%, 3(94/94)	MD 18.01, 95% CI: 14.91 to 21.12, *I* ^2 ^= 75%, 4(173/171)
		Not included	SMD –0.86, 95% CI: –1.13 to –0.59, *I* ^2 ^= 16%, 4(146/138)^d^	SMD –0.50, 95% CI: –0.92 to –0.08, *I* ^2 ^= 0%, 2(44/44)^d^	MD 3.79, 95% CI: –0.33 to 7.91, *I* ^2 ^= 70%, 2(58/60)^c^

BI, Barthel Index; BS, Bai Shao (Paeonia lactiflora Pall.); CHM, Chinese herbal medicine; DG, Dang Gui (Angelica sinensis (Oliv.) Diels); FMA, Fugl-Meyer Assessment; HH, Hong Hua (Carthamus tinctorius L.); (M)AS, (Modified) Ashworth Scale; MG, Mu Gua (Chaenomeles speciosa (Sweet) Nakai); N/A, not applicable; SJC, Shen Jin Cao (Lycopodium japonicum Thunb.). ^a^Results were presented in the way of “SMD, 95% CI, I^2^, No. of studies (No. of participants of I/C groups)”; ^b^Results were presented in the way of “MD, 95% CI, I^2^, No. of studies (No. of participants of I/C groups)”; ^c^No statistically significant difference; ^d^Heterogeneity reduced.

Sensitivity analysis of studies with low risk of bias for sequence generation showed that the significant treatment effects remained, while heterogeneity reduced for the score changes for upper-limb AS or MAS (six studies: Shen et al., 2007; Li et al., 2008; Chen et al., 2010; Ding, 2016; Lai, 2016; Zhang, 2016; SMD −0.86, 95% CI: −1.14 to −0.58, I^2^ = 50%) (**Table 5**), as well as that of lower-limb (three studies: Ou et al., 2007; Shen et al., 2007; Chen et al., 2010; SMD −0.61, 95% CI: −0.96 to −0.27, *I* ^2^ = 0%) (**Table 5**).

### Publication Bias

None of the above meta-analyses included more than 10 trials; therefore, publications bias was not evaluated.

### Herb Analysis

*Bai Shao* (*Paeonia lactiflora* Pall.) was the most frequently used oral herb, reported by 17 studies, followed by *Gan Cao* (*Glycyrrhiza uralensis* Fisch.) (**Table 7**). *Shen Jin Cao* (*Lycopodium japonicum* Thunb.) and *Dang Gui* [*Angelica sinensis* (Oliv.) Diels] were among the most frequently reported topical herbs in the included studies (**Table 7**). In fact, the combination of *Bai Shao* and *Gan Cao* is a traditional oral CHM formula termed *Shao Yao Gan Cao Tang* (SYGCT), which was reported to have anti-spasticity activity (Zhang et al., 2015).

**Table 7:** Frequently used herbs.

	Herbs (Chinese *Pin Yin*)	Academic names	Frequency
Oral herbs	*Bai Shao*	*Paeonia lactiflora Pall.*	17
	*Gan Cao*	*Glycyrrhiza uralensis Fisch.*	13
	*Dang Gui*	*Angelica sinensis (Oliv.) Diels*	12
	*QuanXie*	*Buthus martensii Karsch*	10
	*Di Long*	*Pheretima aspergillum (E.Perrier) or Pheretima vulgaris Chen or Pheretima guillelmi (Michaelsen) or Pheretima pectinifera Michaeken*	9
	*Mu Gua*	*Chaenomeles speciosa (Sweet) Nakai*	9
	*Ji XueTeng*	*Spatholobus suberectus Dunn*	8
	*Shen Jin Cao*	*Lycopodium japonicum Thunb.*	7
	*Huang Qi*	*Astragalus membranaceus (Fisch.) Bge. var. mongholicus (Bge.) Hsiao or Astragalus membranaceus (Fisch.) Bge.*	7
	*Tao Ren*	*Prunuspersica (L.) Batsch or Prunus davidiana (Carr.) Franch*	6
	*Hong Hua*	*Carthamus tinctorius L.*	6
Topical herbs	*Shen Jin Cao*	*Lycopodium japonicum Thunb.*	13
	*Dang Gui*	*Angelica sinensis (Oliv.) Diels*	13
	*Bai Shao*	*Paeonia lactiflora Pall.*	12
	*Hong Hua*	*Carthamus tinctorius L.*	12
	*Mu Gua*	*Chaenomeles speciosa (Sweet) Nakai*	11
	*GuiZhi*	*Cinnamomum cassia Presl*	9
	*Dan Shen*	*Salvia miltiorrhiza Bge.*	9
	*Gan Cao*	*Glycyrrhiza uralensis Fisch.*	9
	*ChuanXiong*	*Ligusticum chuanxiong Hort.*	8
	*Ji XueTeng*	*Spatholobus suberectus Dunn*	7
	*Wei Ling Xian*	*Clematis chinensis Osbeck or Clematis hexapetala Pall. or Clematis manshurica Rupr.*	7

For topical herbs, *post hoc* subgroup analysis was conducted to estimate the effects of individual and the combination of the top five most frequently reported herbal ingredients in the included studies. **Table 6** summarizes the results with significant between-subgroup differences in *post hoc* analysis of herbs. Superior effects were detected in the subgroup of studies in which the formulas included *Bai Shao* than in subgroups of studies without *Bai Shao*, in terms of AS or MAS for the lower limbs (SMD −1.55, 95% CI: −2.29 to −0.82, *I* ^2^ = 79%) and BI (MD 18.01, 95% CI: 14.91 to 21.12, *I* ^2^ = 75%). Similarly, subgroups of studies using *Dang Gui* might have greater benefits than those without this herb, in terms of upper-limb AS or MAS (SMD −1.21, 95% CI: −1.58 to −0.83, *I* ^2^ = 71%) and BI (MD 18.01, 95% CI: 14.91 to 21.12, *I* ^2^ = 75%). It is worth noting that formulas containing three ingredients (*Bai Shao*, *Dang Gui*, and *Hong Hua*) demonstrated a trend for greater efficacy in terms of the three outcome measures: AS or MAS in upper limbs (SMD −1.56, 95% CI: −1.94 to −1.17, *I* ^2^ = 43%), AS or MAS in lower limbs (SMD −1.93, 95% CI: −2.34 to −1.53, *I* ^2^ = 0%), and BI (MD 18.01, 95% CI: 14.91 to 21.12, *I* ^2^ = 75%) than the formulas without these herbs, with reduced heterogeneity.

## Discussion

The results of this systematic review suggested that adding oral or topical CHM to RC for PSS is beneficial for reducing muscle spasticity in the upper and lower extremities. For the overall and lower-limb motor score of FMA and BI, significant add-on effects were observed for both oral and topical CHM. In contrast, no significant effects were seen when adding oral CHM to RC for upper-limb motor function. Mild self-healing adverse events were reported in the intervention group receiving topical CHM; the connections of the CHMs to the adverse events had, however, not been explored.

### Clinical Implications

In our analyses, the changes in AS or MAS scores were merged for analysis using SMD; therefore, the minimum detectable difference or minimum clinically important difference (MCID) was not applied to its clinical interpretation (**Figure 3**). With regard to upper-extremity FMA, both minimum detectable difference and MCID were found to be 5.2 (Wagner et al., 2008; Page et al., 2012). MCID for overall, upper-limb, and lower-limb FMA was found to be 6.0, 4.58, and 3.31, respectively, in another study (Chen et al., 2015). In fact, the changes in the total motor, upper-limb, and lower-limb FMA scores in intervention groups (oral or topical CHM plus RC) and control groups (RC alone) were all greater than the MCID (**Figure 4**). In terms of BI, the minimum detectable difference (4.02 points) (Hsieh et al., 2007) was established and used for interpretation of our results. The use of oral CHM with RC demonstrated clinical advantages for PSS in terms of BI when compared to RC alone (**Figure 5**). The reasons for inconsistent effects among different outcomes should be cautiously interpreted: first, the relatively small numbers of participants and included studies and high heterogeneity limited our confidence in these results; second, a decrease in spasticity severity might not necessarily lead to improvement in motor function (Li, 2017); third, other factors, such as muscle strength, might also contribute to changes in the results, particularly motor function and activities of daily living (Langhammer et al., 2007; Harvey, 2015; Nunes et al., 2016).

This review attempted to explore the characteristics of PSS patients who would benefit from adding CHM therapies to RC, such as the time of stroke onset and the post-stroke period. The results of subgroup analyses suggested that patients with spasticity within 180 days post-stroke might benefit more from additional topical CHM treatment. As for specific CHM treatment, further exploration of potential formulas was not applicable because of the diversity of formulas used in the included studies (**Table 2**). Therefore, we summarized the most frequently reported herbs and conducted subgroup analysis for individual and combinations of herbal ingredients used in the included studies. For oral CHM, *Bai Shao* and *Gan Cao* were the most frequently used herbs, although a subgroup analysis supporting the use of these two herbs was not possible. In terms of topical CHM, a combination of *Bai Shao*, *Dang Gui*, and *Hong Hua* demonstrated a promising therapeutic add-on effect for spasticity reduction and an improvement in activities of daily living. Specifically, for the preparation of topical CHM, steaming therapy with CHM showed a trend for better improvement than CHM compression therapy (**Table 6**). It is worth noting that confounding variables might also have an impact on the results, due to the complexity of the application of topical CHM. For instance, the overall treatment effects of steaming may be a combined result of CHM, steaming water, and heat. Therefore, to distinguish and confirm individual therapeutic efficacy of CHM requires further assessment. Treatment duration was reported to range from 20 days to 3 months among the included trials (**Table 2**). However, subgroup analysis of treatment with a pre-defined cutoff of 4 weeks’ duration was not applicable. Moreover, all participants enrolled in the included studies had already developed spasticity, with AS or MAS ≥ 1. Thus, the effects of CHM on patients at a very early post-stroke stage, when spasticity is not yet detectable with MAS, cannot be known based on the results of this review. Furthermore, all participants enrolled in the included trials were Chinese, and thus the generalizability of the results is not known; additional evidence of using CHM therapy on a non-Chinese population is therefore required.

### Potential Pharmacological Mechanisms

Neuroprotective activity, exerted *via* activation of the adenosine A1 receptor, was observed with paeoniflorin extracted from *Bai Shao* (Liu et al., 2005; Zhang et al., 2009; Tang et al., 2010; Zhang et al., 2017). In terms of *Gan Cao*, potential neuroprotection by one of its major ingredients, glycyrrhizin, was mediated by anti-inflammatory effects *via* inhibition of HMGB1 secretion and inhibition of neurotoxicity by suppression of glutamate-induced apoptosis (Kim et al., 2012b). Triterpene saponins and Licochalcone E in *Gan Cao* were observed to have protective effects against neurotoxicity through suppression of glutamate-induced apoptosis (Cherng et al., 2006; Hwang et al., 2006) and activation of the Nrf2/antioxidant-response element signaling pathway (Kim et al., 2012a). Another bioactive component, Licochalcone A, was shown to have anti-spasmodic activity alone and when combined with paeoniflorin, potentially through inhibition of phosphodiesterases (Sato et al., 2006; Nagai et al., 2007) and by decreasing excitatory amino acid content, respectively (Kimura et al., 1984; Zhang et al., 2015). The ingredients with anti-neurotoxicity effects in *Dang Gui* include polysaccharides, organic acids, and phthalides. Potential mechanisms include decreased expression of nicotinic acetylcholine receptors (Gu et al., 2008) and increased brain-derived neurotrophic factor and nerve growth factor protein expression (Chen et al., 2009). Similarly, neuroprotective function could also be observed for ingredients of *Hong Hua* (He et al., 2012; Yu et al., 2013; Zhang et al., 2016). A combination of topically used *Dang Gui*, *Hong Hua*, and *Bai Shao* demonstrated a promising benefit for PSS (**Table 6**), but the underlying mechanism is yet to be unveiled. Representative examples of major neurological effects and potential mechanisms are summarized in **Table 8**.

**Table 8:** Representative examples of major neurological effects and potential mechanisms.

Herbs	Bioactive ingredients	Related formulations	Beneficial effects	Potential mechanisms	Experimental models	Ref
*Dang Gui (Angelica sinensis (Oliv). Diels)*	1) Polysaccharides 2) Organic acids 3) Phthalides	1) *Jia Wei Bu Yang Huan Wu Tang* 2) *Tong Luo Jie Jing Tang* 3) *Shu JinHuo Luo Xi Ji*	Neuroprotective effects	Increasing brain-derived neurotrophic factor and nerve growth factor protein expression	Rats	Nunes et al., 2016
			Inhibit neurotoxicity	Decreased expression of nicotinic acetylcholine receptors induced by β-amyloid protein	Human neuroblastoma cells	Langhammer et al., 2007
*Bai Shao (Paeonialactiflora Pall).*	Paeoniflorin	1) *Shao Yao Gan Cao Tang* 2) *Gua Lou GuiZhi Tang* 3) *Tong Luo Jie Jing Tang* 4) *Jie Jing He Ji*	Neuroprotective activity	Activating adenosine A1 receptor: 1) scavenging superoxide anions, inhibiting microglial activation and IL-1β, NF-κB, TNF-α expressions 2) attenuated neuronal apoptosis by regulating the Ca^2+^/CaMKII/CREB signaling pathway	Rats	Liu et al., 2005, Tang et al., 2010, Zhang et al., 2017
			Anti-spasmodic activity	(Combined with paeoniflorin and glycyrrhizin): 1) decrease excitatory amino acids content 2) inhibit muscle contraction	1) Frogs and mice 2) Rats	Kimura et al., 1984, Gu et al., 2008
			Analgesic activity	*Paeoniflorin* (180 mg/kg): inhibiting the extracellular signal-regulated protein kinase (ERK) pathway	Rats	Zhang et al., 2009
*Gan Cao (Glycyrrhizauralensis Fisch).*	Glycyrrhizin (glycyrrhizic acid)	1) *Shao Yao Gan Cao Tang* 2) *Gua Lou GuiZhi Tang* 3) *Shu Jin Tong Luo Fang* 4) *Jie Jing He Ji*	Neuroprotective effects	Anti-inflammatory effects by inhibiting HMGB1 secretion, anti-excitotoxic, and anti-oxidative	Rats	Kim et al., 2012b
*Gan Cao (Glycyrrhizauralensis Fisch).*	Triterpene saponins	1) *Shao Yao Gan Cao Tang* 2) *Gua Lou GuiZhiTang* 3) *Shu Jin Tong Luo Fang* 4) *Jie Jing He Ji*	Inhibit neurotoxicity	Suppression of the glutamate-induced apoptosis by: 1) inhibiting the Ca^2+^ influx activated through NMDA receptor by glutamate 2) diminishing DNA fragmentation and cleavage of PARP 3) inhibiting the binding activity of NF-κb 4) maintaining the SOD1 levels	Rat neuronal cultures and merionesunguiculatus	Cherng et al., 2006, Hwang et al., 2006
	LicochalconeA		Anti-spasmodic activity	Inhibit PDEs, especially isozyme 3, followed by the accumulation of intracellular cAMP	Mouse jejunum	Nagai et al., 2007, Sato et al., 2006
	LicochalconeE		Neuroprotection	Activates Nrf2/antioxidant response element signaling pathway	Mouse cells	Kim et al., 2012a
*Hong Hua (Carthamustinctorius L.).*	Hydroxysaffloryellow A	1) *Bu Yang Huan Wu Tang* 2) *Yi Qi Rou Jin Tang* 3) *Shu JinHuo Luo Xi Ji*	Neuroprotective function	Suppression of apoptosis by the regulation of Bcl-2 and Bax protein expression	Rats	Yu et al., 2013
	Kaempferol-3-O-rutinoside		Prevent ischemic brain injury and inflammation	Inhibit the activation of NF-κB and STAT3	Rats	Chen et al., 2009
*Mu Gua (Chaenomelesspeciosa (Sweet) Nakai)*	Oleanolic acid Ursolic acid	1) *Tong Luo Jie Jing Tang* 2) *Rou Jin Tang* 3) *Shu JinHuo Luo Xi Ji* 4) *JieJin He Ji*	Inhibit neurotoxicity	Inhibit neuronal death by elevating intracellular Ca^2+^ concentration, and generation of ROS	Rat cortical neurons	Zhang et al., 2016

Bax protein, Bcl-2-associated X protein; Bcl-2, B-cell lymphoma 2; Ca^2+^, calcium ion; CaMKII, calmodulin-dependent protein kinase II; cAMP, Cyclic adenosine monophosphate; CREB, cAMP response element-binding; HMGB1, high mobility group box1protein; IL-1β, interleukin-1beta; NF-κB, nuclear factor-kappa B; NMDA, N-methyl-D-aspartate; Nrf2, nuclear factor E2-related factor 2; PARP, poly-ADP-ribose polymerase; PDE, phosphodiesterase; Ref, references; ROS, reactive oxygen species; SOD1, superoxide dismutase 1; STAT3, signal transducer and activator of transcription 3; TNF-α, tumor necrosis factor-alpha. Australian National Stroke Foundation (2017).

However, various formulas with complex compounds were used in the included studies, and the potentially active ingredients isolated from CHM usually act on different mechanisms and pathways. There is no direct research evidence from studies on human skeletal muscles using these bioactive ingredients to exploring the underlying mechanisms of the effects on spasticity specifically; spasticity is characterized by a velocity-dependent increase in tonic stretch reflexes (Lance, 1980). Therefore, the causal relationship between the observed therapeutic effects on PSS and the individual components or monomolecular substance targeting at a few known cellular or molecular pathways could not be confirmed. Further mechanistic and clinical studies are needed to elucidate how the bioactive CHM ingredients work individually and interactively, to optimize and even standardize CHM components and treatment protocols in future.

### Limitations

Safety and long-term tolerance of therapy are a concern in the treatment of PSS (Nair and Marsden, 2014). Our systematic review suggested that oral and topical CHM were well tolerated during a treatment period as long as 3 months, with mild adverse events among 10 studies (Zhu et al., 2002; Shen et al., 2007; Zhu et al., 2007; Zhang, 2009; Chen et al., 2010; Huang, 2011; Zhao, 2013; Ou et al., 2014; Weng, 2014; Lai, 2016) (**Table 1**). However, the remaining 25 studies did not address the safety issue and none of the included studies covered a follow-up period, making the assessment of long-term safety inapplicable based on the results of our systematic review.

Proper randomization and allocation are essential for reducing selection bias in RCTs. However, in this systematic review, only 51.4% of the included studies applied appropriate methods for sequence generation, and only 5.7% did so for allocation concealment (**Figure 2**). Both of these deficits might lead to underestimation or overestimation of the treatment effects (Pildal et al., 2007). It is worth noting that none of the included trials attempted to blind participants or personnel with the use of an appropriate placebo, and outcome assessors were blinded in only three studies (Zhang et al., 2007; Liu et al., 2014a; Weng, 2014). Admittedly, there is no easy way to perform double-blinding with oral or topical CHM therapies, whose preparation, appearance, taste, and smell are so diverse that placebo control might be difficult. In the context of this challenge with decoction, other forms of oral CHM could be considered if applicable, such as granule, capsule, or dropping pills. In addition, given the improvement in the preparation of CHM and the extraction technique of active components, lipophilic compounds of herbs, such as Tanshinone, that could not be efficiently extracted through traditional decoction, might be available with supercritical carbon dioxide (Esquivel-Hernandez et al., 2016; Sulniute et al., 2017). With such techniques, the effective compounds can be extracted more efficiently, and the quality control of CHM products can be improved. Moreover, the use of more advanced CHM products may make the double-blinded, placebo-controlled trial design feasible. Furthermore, tests of blinding with placebo are needed before conducting a randomized control trial, and evaluation of addition of CHM efficacy as compared with placebo, added to rehabilitation therapies or pharmacotherapies, are required, especially for non-objective outcome assessments.

Another limitation of the synthesis results is the reporting quality of the included studies. None of the included studies reported all key items recommended by CONSORT 2010 and its Extension for Herbal Intervention and Chinese Herbal Medicine Formulas (Gagnier et al., 2006; Schulz et al., 2010; Cheng et al., 2017). Even among those reported in original studies, ambiguous terms were frequently seen (**Table 2**). For example, instead of specific volume, “dose” was frequently used in the reporting of oral CHM interventions. The reported oral solutions in the included studies are difficult to distinguish clearly from decoction, whose scope is yet to be specifically defined. Therefore, future trials need to improve reporting quality, and specific definitions and standardization of CHM interventions require further research and agreement.

### Disagreement With Existing Reviews

We identified one published systematic review and meta-analysis investigating the effects of the oral CHM formula SYGCT for PSS (Chen and Tan, 2016). Ten RCTs involving 732 participants were included in that meta-analysis through a database search from January 1990 to November 2015. The Jadad scale was used to assess the methodological quality of the included studies. Based on the synthesis results of FMA and BI, the review concluded that the decoction SYGCT had potential benefits for patients with PSS. However, because different comparisons, such as SYGCT vs. RC, and SYGCT add-on to RC vs. RC, were pooled into one meta-analysis, this conclusion was not confirmed. Moreover, that review did not evaluate the outcome related to the severity of spasticity. Our systematic review and meta-analysis differed from this previous systematic review in the following ways: First, our review focused specifically on the add-on effects of CHM, including oral and topical CHM for PSS; second, comprehensive outcome measures were evaluated in terms of spasticity severity, motor function, and activities of daily living; third, our research provided up-to-date evidence by performing a search from database inception to February 2018; fourth, the Cochrane risk-of-bias tool was used for methodological quality assessment, since the validity of the total score of the Jadad scale has increasingly been challenged (Emerson et al., 1990; Schulz et al., 1995; Juni et al., 1999).

## Conclusions

Within the limitations of the quality concerns of the included trials, this review suggested that CHM is a well-tolerated potential add-on therapy for patients with PSS. Future trials of high methodological quality with prospectively registered protocols and valid placebo control are needed to confirm the add-on effectiveness of CHM in reducing spasticity and improving daily activities.

## Author’s Note

Protocol registration information: PROSPERO 2016: CRD42016043281. Available: https://www.crd.york.ac.uk/PROSPERO/display_record.php?RecordID=43281

## Author Contributions

CL and CX initiated the research. YC, CZ, and SL conducted the database search, study screening, data extraction, and data analyses. AZ, ZW, and XG were involved in data analysis and interpretation and in resolving disagreements. YC and CZ drafted the manuscript. All authors contributed to manuscript revision and read and approved the submitted version.

## Funding

This work was supported by 1) “Construction of High-level University” Public Projects from Guangzhou University of Chinese Medicine [no. (2016) 64]; 2) “Specific Research Fund for TCM Science and Technology of Guangdong Provincial Hospital of Chinese Medicine (2016)” (no. YN2016QL01); 3) “National Key Technology R&D Program for the 12th Five-year Plan of Ministry of Science and Technology, China” (no. 2013BAI02B10); and 4) China–Australia International Research Centre for Chinese Medicine, funded by the Guangdong Provincial Academy of Chinese Medical Sciences and Guangdong Provincial Hospital of Chinese Medicine, Guangdong, China, and RMIT University, Australia. The funders had no role in the study design, data collection and analysis, decision to publish, or preparation of the manuscript. Authors’ positions are partially supported by the above funding.

## Conflict of Interest Statement

The authors declare that the research was conducted in the absence of any commercial or financial relationships that could be construed as a potential conflict of interest.

## Abbreviations

AS, Ashworth Scale; BI, Barthel Index; CHM, Chinese herbal medicine; CI, confidence interval; FMA, Fugl-Meyer Assessment; MAS, Modified Ashworth Scale; MCID, minimum clinically important difference; MD, mean difference; PSS, post-stroke spasticity; RC, routine care; RCT(s), randomized controlled trial(s); SD, standard difference; SMD, standard mean difference; SYGCT, *Shao Yao Gan Cao Tang*.
