# Supplementary material for: Add-On Effects of Chinese Herbal Medicine for Post-Stroke Spasticity: A Systematic Review and Meta-Analysis
Source: Front Pharmacol. 2019 Jun 27;10:734. doi: 10.3389/fphar.2019.00734 (PMC6610255; doi:10.3389/fphar.2019.00734)
Supplement: Supplementary file 1 [file Table_1.docx]

Table S1 Search strategy

|  | Search | Search strategy |
| --- | --- | --- |
| English databases | #1 | stroke or brain infarction or intracranial embolism and thrombosis or intracranial hemorrhages or cerebrovascular accident or cerebral infarction or intracranial haemorrhage or cerebral |
|  | #2 | muscle spasticity or muscle hypertonia or muscle tonus or spasm or dystonia or paraparesis, spastic* or high tone or hypermyotonia or contracture |
|  | #3 | traditional Chinese medicine or Chinese traditional medicine or Chinese herbal drugs or Chinese drugs, plant or medicine, traditional or ethnopharmacology or ethnomedicine or ethnobotany or medicine, medicinal or plant preparation or plant extract or plants, medicine or materia medica or single prescription or herbs or Chinese medicine herb or herbal medicine |
|  | #4 | random* and controlled trial random* and controlled trial |
|  | #5 | #1 and #2 and #3 and #4 |
| Chinese databases | #6 | 颅内栓塞和血栓形成or颅内栓塞or颅内血栓形成or脑梗死or脑干梗死or颅内出血or脑出血or蛛网膜下腔出血or急性脑血管病or卒中or脑血管意外or脑溢血or脑梗塞or脑血栓or缺血性脑血管病 |
|  | #7 | 偏瘫or痉挛or关节挛缩or肌张力障碍or肌无力or瘫痪or半身不遂or康复or运动疗法or功能锻炼or功能训练 |
|  | #8 | 中医 or 中西医 or 中医疗法 or 辨证 or 祖国医学 or 传统治疗 or 传统疗法 or 替代医学 or 替代治疗 or 中国传统医学 or 草药 or 中草药 or 中药 or 中药疗法 or 中西药 or 传统医药 or 中成药 or 植物药 or 治则 or 熏洗 or 薰洗 or 药浴 or 外洗 or 浴足 or 灌肠 or 药熨 or 热敷 or 足疗 OR雾化 or 中药外敷 or 外敷 OR熏蒸 OR薰蒸 |
|  | #9 | 临床观察 or 临床评估 or 临床试验 or 临床效果 or 临床研究 or 疗效 or 评价研究 or 前瞻性 or 随访 or 对比研究 or 多中心 or 随机 or 对照 or 病例报告 or 病例研究 or 病例分析 or 病例报道 |
|  | #10 | #6 and #7 and #8 and #9 |
